# Supplementary figures and images for: A mathematical model assuming frequency-dependent cost for analyzing the influence of cell competition on radiation effects
Source: PLoS One. 2025 Nov 21;20(11):e0337111. doi: 10.1371/journal.pone.0337111 (PMC12637972; doi:10.1371/journal.pone.0337111)

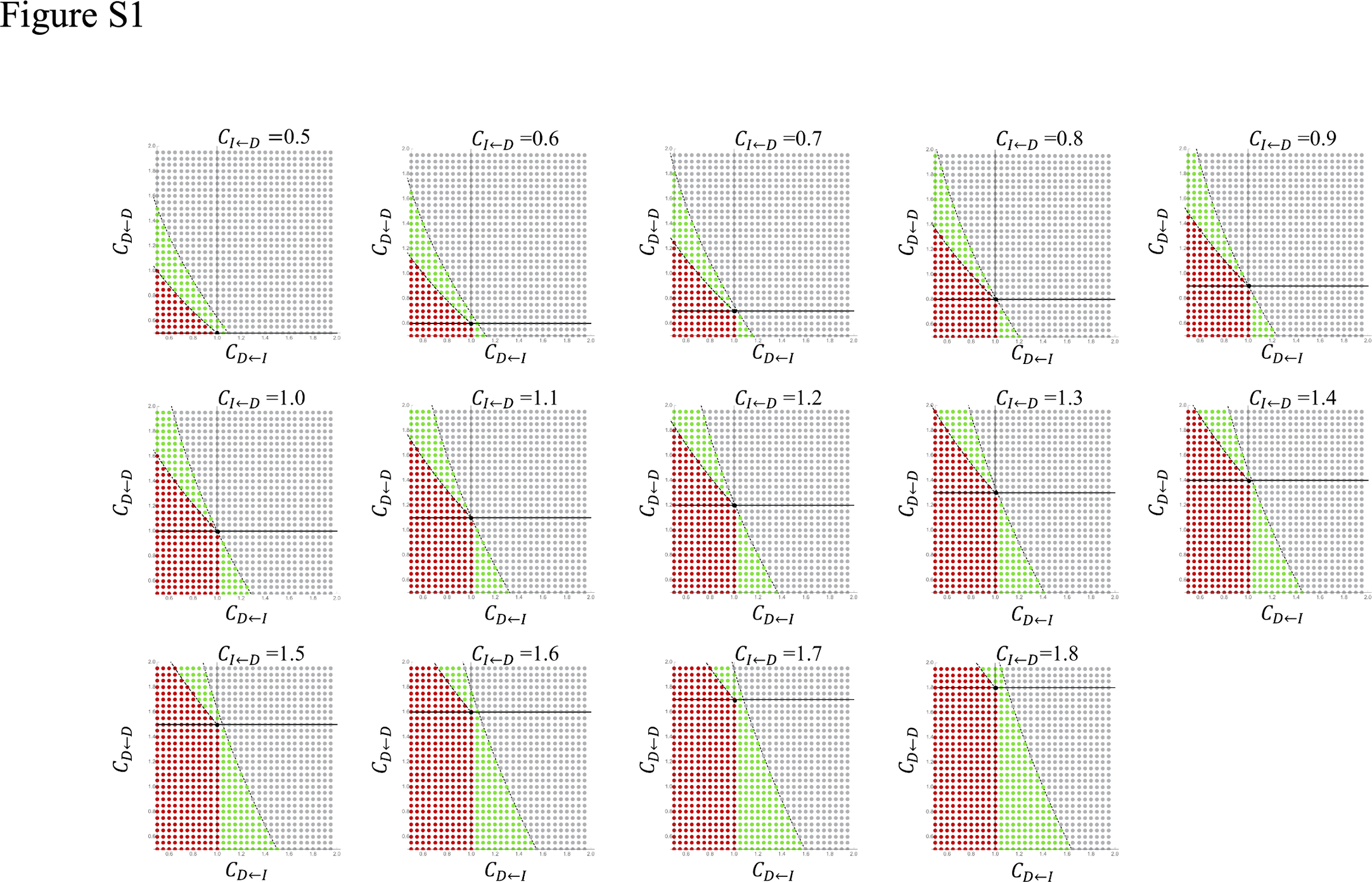

Supplement: S1 Fig — The parameter CI←I is fixed as CI←I=1 without loss of generality. The gray, red, and green dots represent monotonic increase, monotonic decrease, and convex downward, respectively. The black dot represents the case that Tabs did not change depending on N.The vertical and horizontal lines in the diagram represent CD←I=CI←I and CD←D=CI←D, respectively. The dotted line represents the decrease in parameter Tabs when N changes from N=2 to N=3. The dotted-dashed line represents the increase in parameter Tabs when N is very large (here, N=105). (TIF) [file pone.0337111.s001.tif]
