# Supplementary material for: A mathematical model assuming frequency-dependent cost for analyzing the influence of cell competition on radiation effects
Source: PLoS One. 2025 Nov 21;20(11):e0337111. doi: 10.1371/journal.pone.0337111 (PMC12637972; doi:10.1371/journal.pone.0337111)
Supplement: S1 File — (DOCX) [file pone.0337111.s002.docx]

Supporting information

**Appendix A**

*Absorbing state of the model*

Let $D_{i}\left( s \right)$ denote probabilities that the number of damaged cells in the cell pool is $i$ after $s$ cycles of stochastic transition, cell division, and elimination of a cell. Well-mix model is written as follows, see also [20]:

$$\left[ \begin{matrix} D_{0}\left( s+1 \right) \\ D_{1}\left( s+1 \right) \\ \vdots\\ D_{N}\left( s+1 \right) \end{matrix} \right]=QP\left[ \begin{matrix} D_{0}\left( s \right) \\ D_{1}\left( s \right) \\ \vdots\\ D_{N}\left( s \right) \end{matrix} \right],$$

where

$P=\left[ \begin{matrix} 1-H & 0 & 0 & \cdots& 0 & 0 & 0 \\ H & 1-\frac{\left( N-1 \right)H}{N} & 0 & \cdots& 0 & 0 & 0 \\ 0 & \frac{\left( N-1 \right)H}{N} & 1-\frac{\left( N-2 \right)H}{N} & \cdots& 0 & 0 & 0 \\ \vdots& \vdots& \vdots& \ddots& \vdots& \vdots& \vdots\\ 0 & 0 & 0 & \cdots& 1-\frac{2H}{N} & 0 & 0 \\ 0 & 0 & 0 & \cdots& \frac{2H}{N} & 1-\frac{H}{N} & 0 \\ 0 & 0 & 0 & \cdots& 0 & \frac{H}{N} & 1 \end{matrix} \right]^{r},$

$Q=\left[ \begin{matrix} P_{0,0} & P_{1,0} & P_{2,0} & \cdots& P_{N-2,0} & P_{N-1,0} & P_{N,0} \\ P_{0,1} & P_{1,1} & P_{2,1} & \cdots& P_{N-2,1} & P_{N-1,1} & P_{N,1} \\ P_{0,2} & P_{1,2} & P_{2,2} & \cdots& P_{N-2,2} & P_{N-1,2} & P_{N,2} \\ \vdots& \vdots& \vdots& \ddots& \vdots& \vdots& \vdots\\ P_{0,N-2} & P_{1,N-2} & P_{2,N-2} & \cdots& P_{N-2,N-2} & P_{N-1,N-2} & P_{N,N-2} \\ P_{0,N-1} & P_{1,N-1} & P_{2,N-1} & \cdots& P_{N-2,N-1} & P_{N-1,N-1} & P_{N,N-1} \\ P_{0,N} & P_{1,N} & P_{2,N} & \cdots& P_{N-2,N} & P_{N-1,N} & P_{N,N} \end{matrix} \right].$

The matrix $P$ represents stochastic transition, and the matrix $Q$ represents cell division and elimination. The entry of $Q$, $P_{i,j}$, are the probabilities that the number of damaged cells changes from $i$ to $j$ through cell division and elimination. The matrix product QP has a column sums equal to 1, with the entry at $\left( N,N \right)$ equal to 1 and all other entries are greater than or equal to 0 and less than 1. This means that once the cell pool is occupied by damaged cells, intact cells will never arise again. Therefore, the occupancy of the cell pool by damaged cells is the unique absorbing state.

Appendix B

Calculation of $\boldsymbol{\pi}_{\boldsymbol{1}}$

Let $\pi_{i}$ be the probability that damaged cells occupy the cell pool when starting from $i$ damaged cells without transitions. Note that $\pi_{N}=1 \mathrm{and}$ $\pi_{0}=0$, $\pi_{i}$ can be written as follows:

$$\pi_{i}=\sum_{m=0}^{N} P_{i,m}\pi_{m}. \left( A1 \right)$$

$P_{i,j}$ is the probability that the number of damaged cells changes from $i$ to $j$ through cell division and elimination, as shown in Eq. (3) in the main text. When $1\leq i\leq N-1$ Substituting the Eq. (3),

$$\pi_{i}=P_{i,i+1}\pi_{i+1}+P_{i,i}\pi_{i}+P_{i,i-1}\pi_{i-1}$$

$$=P_{i,i+1}\pi_{i+1}+\left( 1-P_{i,i+1}-P_{i,i-1} \right)\pi_{i}+P_{i,i-1}\pi_{i-1}$$

$$\to P_{i,i+1}\left( \pi_{i+1}-\pi_{i} \right)=P_{i,i-1}\left( \pi_{i}-\pi_{i-1} \right)$$

$$\to\pi_{i+1}-\pi_{i}=\frac{P_{i,i-1}}{P_{i,i+1}}\left( \pi_{i}-\pi_{i-1} \right)$$

$$=\frac{\frac{N-i}{N}\frac{iG_{i}}{\left( N-i \right)F_{i}+kG_{i}}}{\frac{i}{N}\frac{\left( N-i \right)F_{i}}{\left( N-i \right)F_{i}+kG_{i}}}\left( \pi_{i}-\pi_{i-1} \right)$$

$$=\frac{G_{i}}{F_{i}}\left( \pi_{i}-\pi_{i-1} \right)=\frac{G_{i}}{F_{i}}\frac{G_{i-1}}{F_{i-1}}\left( \pi_{i-1}-\pi_{i-2} \right)=\ldots=\prod_{x=1}^{i} \frac{G_{x}}{F_{x}}\left( \pi_{1}-\pi_{0} \right). \left( A2 \right)$$

Taking the sum of $\pi_{i+1}-\pi_{i}$ from $i=1$ to $i=N-1,$

$$\pi_{N}-\pi_{1}=\left( \pi_{1}-\pi_{0} \right)\left( \sum_{i=1}^{N-1} \prod_{x=1}^{i} \frac{G_{x}}{F_{x}} \right)\to1-\pi_{1}=\pi_{1}\left( \sum_{i=1}^{N-1} \prod_{x=1}^{i} \frac{G_{x}}{F_{x}} \right)\to\pi_{1}=\frac{1}{1+\sum_{i=1}^{N-1} \prod_{x=1}^{i} \frac{G_{x}}{F_{x}}} \left( A3 \right).$$

This equals to Eq. $\left( 7b \right).$

Appendix C

Boundaries of the shape of $\boldsymbol{T}_{\boldsymbol{abs}}$

To clarify $T_{abs}$ as a function of $N$, we denote it as $T_{abs}\left( N \right)$. According to Eq. (7a) in the main text, $T_{abs}\left( N \right)$ is

$$T_{abs}\left( N \right)=\frac{1}{N\lambda\pi_{1}}=\frac{1+\sum_{j=1}^{N-1} \prod_{k=1}^{j} \frac{G_{x}}{F_{x}}}{N\lambda}=\frac{1+\sum_{j=1}^{N-1} \prod_{k=1}^{j} \frac{C_{D\leftarrow I}\left( N-k \right)+C_{D\leftarrow D}\left( k-1 \right)}{C_{I\leftarrow I}\left( N-k-1 \right)+C_{I\leftarrow D}k}}{N\lambda}, \left( B1 \right)$$

First, we calculate the change of $T_{abs}\left( N \right)$ when $N$ changes from $N=2$ to $N=3$.

$$T_{abs}\left( 3 \right)-T_{abs}\left( 2 \right)=\frac{1+\frac{2C_{D\leftarrow I}}{C_{I\leftarrow I}+C_{I\leftarrow D}}+\frac{2C_{D\leftarrow I}}{C_{I\leftarrow I}+C_{I\leftarrow D}}\frac{C_{D\leftarrow I}+C_{D\leftarrow D}}{2C_{I\leftarrow D}}}{3\lambda}-\frac{1+\frac{C_{D\leftarrow I}}{C_{I\leftarrow D}}}{2\lambda}=\frac{1}{6\lambda C_{I\leftarrow D}\left( C_{I\leftarrow I}+C_{I\leftarrow D} \right)}\left\{ \left( -C_{I\leftarrow D}-3C_{D\leftarrow I} \right)\left( C_{I\leftarrow I}+C_{I\leftarrow D} \right)+2C_{D\leftarrow I}\left( 2C_{I\leftarrow D}+C_{D\leftarrow I}+C_{D\leftarrow D} \right) \right\} \left( B2 \right)$$

When $T_{abs}\left( 3 \right)-T_{abs}\left( 2 \right)=0,$

$$\left( -C_{I\leftarrow D}-3C_{D\leftarrow I} \right)\left( C_{I\leftarrow I}+C_{I\leftarrow D} \right)+2C_{D\leftarrow I}\left( 2C_{I\leftarrow D}+C_{D\leftarrow I}+C_{D\leftarrow D} \right)=0\to C_{D\leftarrow D}=\frac{\left( C_{I\leftarrow D}+3C_{D\leftarrow I} \right)\left( C_{I\leftarrow I}+C_{I\leftarrow D} \right)}{2C_{D\leftarrow I}}-2C_{I\leftarrow D}-C_{D\leftarrow I}. \left( B3 \right)$$

This equation is shown by the dotted line in Figure 5 and Figure S1. Next, we calculated the change in $T_{abs}\left( N \right)$ when $N$ was very large. If $\prod_{k=1}^{N-1} \frac{C_{D\leftarrow I}\left( N-k \right)+C_{D\leftarrow D}\left( k-1 \right)}{C_{I\leftarrow I}\left( N-k-1 \right)+C_{I\leftarrow D}k}$ decreases monotonically depending on $N,$ $\lim_{N\to\infty} T_{abs}\left( N \right)$ converge to 0. Therefore, we calculated the following conditions:

$$\prod_{k=1}^{N-1} \frac{C_{D\leftarrow I}\left( N-k \right)+C_{D\leftarrow D}\left( k-1 \right)}{C_{I\leftarrow I}\left( N-k-1 \right)+C_{I\leftarrow D}k}=\prod_{k=1}^{N} \frac{C_{D\leftarrow I}\left( N+1-k \right)+C_{D\leftarrow D}\left( k-1 \right)}{C_{I\leftarrow I}\left( N+1-k-1 \right)+C_{I\leftarrow D}k}. \left( B4 \right)$$

By expanding and simplifying both sides,

$$\frac{C_{D\leftarrow I}\left( N-1 \right)}{C_{I\leftarrow I}\left( N-2 \right)+C_{I\leftarrow D}}\frac{C_{D\leftarrow I}\left( N-2 \right)+C_{D\leftarrow D}}{C_{I\leftarrow I}\left( N-3 \right)+2C_{I\leftarrow D}}\ldots\frac{C_{D\leftarrow I}+C_{D\leftarrow D}\left( N-2 \right)}{C_{I\leftarrow D}\left( N-1 \right)}=\frac{C_{D\leftarrow I}N}{C_{I\leftarrow I}\left( N-1 \right)+C_{I\leftarrow D}}\frac{C_{D\leftarrow I}\left( N-1 \right)+C_{D\leftarrow D}}{C_{I\leftarrow I}\left( N-2 \right)+2C_{I\leftarrow D}}\ldots\frac{C_{D\leftarrow I}+C_{D\leftarrow D}\left( N-1 \right)}{C_{I\leftarrow D}N}$$

$$\to\frac{C_{I\leftarrow D}}{C_{D\leftarrow I}}\frac{\left\{ C_{D\leftarrow I}\left( N-1 \right) \right\}\left\{ C_{D\leftarrow I}\left( N-2 \right)+C_{D\leftarrow D} \right\}\ldots\left\{ C_{D\leftarrow I}+C_{D\leftarrow D}\left( N-2 \right) \right\}}{\left\{ C_{D\leftarrow I}\left( N-1 \right)+C_{D\leftarrow D} \right\}\left\{ C_{D\leftarrow I}\left( N-2 \right)+2C_{D\leftarrow D} \right\}\ldots\left\{ C_{D\leftarrow I}+C_{D\leftarrow D}\left( N-1 \right) \right\}}=\frac{\left\{ C_{I\leftarrow I}\left( N-2 \right)+C_{I\leftarrow D} \right\}\left\{ C_{I\leftarrow I}\left( N-3 \right)+2C_{I\leftarrow D} \right\}\ldots\left\{ C_{I\leftarrow D}\left( N-1 \right) \right\}}{\left\{ C_{I\leftarrow I}\left( N-1 \right)+C_{I\leftarrow D} \right\}\left\{ C_{I\leftarrow I}\left( N-2 \right)+2C_{I\leftarrow D} \right\}\ldots\left\{ C_{I\leftarrow I}+C_{I\leftarrow D}\left( N-1 \right) \right\}}$$

$$\to\frac{C_{I\leftarrow D}}{C_{D\leftarrow I}}\frac{\left\{ \left( N-1 \right) \right\}\left\{ \left( N-2 \right)+\frac{C_{D\leftarrow D}}{C_{D\leftarrow I}} \right\}\ldots\left\{ 1+\frac{C_{D\leftarrow D}}{C_{D\leftarrow I}}\left( N-2 \right) \right\}}{\left\{ \left( N-1 \right)+\frac{C_{D\leftarrow D}}{C_{D\leftarrow I}} \right\}\left\{ \left( N-2 \right)+2\frac{C_{D\leftarrow D}}{C_{D\leftarrow I}} \right\}\ldots\left\{ 1+\frac{C_{D\leftarrow D}}{C_{D\leftarrow I}}\left( N-1 \right) \right\}}=\frac{\left\{ \frac{C_{I\leftarrow I}}{C_{I\leftarrow D}}\left( N-2 \right)+1 \right\}\left\{ \frac{C_{I\leftarrow I}}{C_{I\leftarrow D}}\left( N-3 \right)+2 \right\}\ldots\left\{ \left( N-1 \right) \right\}}{\left\{ \frac{C_{I\leftarrow I}}{C_{I\leftarrow D}}\left( N-1 \right)+1 \right\}\left\{ \frac{C_{I\leftarrow I}}{C_{I\leftarrow D}}\left( N-2 \right)+2 \right\}\ldots\left\{ \frac{C_{I\leftarrow I}}{C_{I\leftarrow D}}+\left( N-1 \right) \right\}} \left( B5 \right)$$

Let $g\left( x \right)=\left( N-x \right)+x\frac{C_{I\leftarrow I}}{C_{I\leftarrow D}}\mathrm{and}f\left( x \right)=\left( N-x \right)+x\frac{C_{D\leftarrow D}}{C_{D\leftarrow I}}$, eq. $\left( B5 \right)$ is

$$\frac{C_{I\leftarrow D}}{C_{D\leftarrow I}}\frac{\left\{ f\left( 1 \right)-\frac{C_{D\leftarrow D}}{C_{D\leftarrow I}} \right\}\left\{ f\left( 2 \right)-\frac{C_{D\leftarrow D}}{C_{D\leftarrow I}} \right\}\ldots\left\{ f\left( N-1 \right)-\frac{C_{D\leftarrow D}}{C_{D\leftarrow I}} \right\}}{f\left( 1 \right)f\left( 2 \right)\ldots f\left( N-1 \right)}=\frac{\left\{ g\left( N-1 \right)-\frac{C_{I\leftarrow I}}{C_{I\leftarrow D}} \right\}\left\{ g\left( N-2 \right)-\frac{C_{I\leftarrow I}}{C_{I\leftarrow D}} \right\}\ldots\left\{ g\left( 1 \right)-\frac{C_{I\leftarrow I}}{C_{I\leftarrow D}} \right\}}{g\left( N-1 \right)g\left( N-2 \right)\ldots g\left( 1 \right)}$$

$$\to\frac{C_{I\leftarrow D}}{C_{D\leftarrow I}}\left\{ 1-\frac{1}{f\left( 1 \right)}\frac{C_{D\leftarrow D}}{C_{D\leftarrow I}} \right\}\left\{ 1-\frac{1}{f\left( 2 \right)}\frac{C_{D\leftarrow D}}{C_{D\leftarrow I}} \right\}\ldots\left\{ 1-\frac{1}{f\left( N-1 \right)}\frac{C_{D\leftarrow D}}{C_{D\leftarrow I}} \right\}=\left\{ 1-\frac{1}{g\left( N-1 \right)}\frac{C_{I\leftarrow I}}{C_{I\leftarrow D}} \right\}\left\{ 1-\frac{1}{g\left( N-2 \right)}\frac{C_{I\leftarrow I}}{C_{I\leftarrow D}} \right\}\ldots\left\{ 1-\frac{1}{g\left( 1 \right)}\frac{C_{I\leftarrow I}}{C_{I\leftarrow D}} \right\}$$

$$\to\frac{C_{I\leftarrow D}}{C_{D\leftarrow I}}\prod_{i=1}^{N} \left\{ 1-\frac{1}{F\left( i \right)} \right\}=\prod_{i=1}^{N} \left\{ 1-\frac{1}{G\left( i \right)} \right\}, \left( B6 \right)$$

where $F\left( i \right)=\left( {C_{D\leftarrow I}}/{C_{D\leftarrow D}} \right)f\left( i \right), G\left( i \right)=\left( {C_{I\leftarrow D}}/{C_{I\leftarrow I}} \right)g\left( i \right)$. The numerical solution when $N={10}^{5}$ is indicated by the dotted-dashed line in Figure 5 and the Figure S1.
