# Supplementary material for: A mathematical model assuming frequency-dependent cost for analyzing the influence of cell competition on radiation effects
Source: PLoS One. 2025 Nov 21;20(11):e0337111. doi: 10.1371/journal.pone.0337111 (PMC12637972; doi:10.1371/journal.pone.0337111)
Supplement: Attachment — Submitted filename: Response_to_Reviewers_auresp_2.docx [file pone.0337111.s005.docx]

Response to Reviewer #2:

>My previous concern that “the dose parameter, \lambda, is not contained in the equations of the

>competition model in equation 3, making it difficult to determine its influence” was not addressed. I

>remain concerned that the model methods still isn't written in such a way that is reproducible.

>Based on the authors' response, i now understand that the Moran process has 3 steps: transition, birth,

>replacement. The equations in 3a, 3b, 3c, and 3d only describe birth and replacement. Please consider

>updating these equations to be explicit functions of lambda, to include the transition step.

[Answer]

The dose rate λ can affect the equations 3a, 3b, 3c, and 3d through cost parameters. However, detailed analyses of this assumption would be complex and be beyond the scope of the present study, so we did not modify the equations. Nevertheless, the reviewer’s comment is biologically important point. We have therefore included a discussion in the Discussion section on how the properties of damaged cells may relate to the λ-dependence of the parameters (Line 501-508).
